# Screening of a Library of Recombinant *Schistosoma mansoni* Proteins With Sera From Murine and Human Controlled Infections Identifies Early Serological Markers

**DOI:** 10.1093/infdis/jiaa329

**Published:** 2020-06-10

**Authors:** Cécile Crosnier, Cornelis H Hokke, Anna V Protasio, Cordelia Brandt, Gabriel Rinaldi, Marijke C C Langenberg, Simon Clare, Jacqueline J Janse, Shona Wilson, Matthew Berriman, Meta Roestenberg, Gavin J Wright

**Affiliations:** 1 Wellcome Sanger Institute, Cambridge, United Kingdom; 2 Department of Parasitology, Leiden University Medical Center, Leiden, the Netherlands; 3 Department of Pathology, University of Cambridge, Cambridge, United Kingdom

**Keywords:** antibodies, *Schistosoma mansoni*, schistosomiasis, serology

## Abstract

**Background:**

Schistosomiasis is a major global health problem caused by blood-dwelling parasitic worms, which is currently tackled primarily by mass administration of the drug praziquantel. Appropriate drug treatment strategies are informed by diagnostics that establish the prevalence and intensity of infection, which, in regions of low transmission, should be highly sensitive.

**Methods:**

To identify sensitive new serological markers of *Schistosoma mansoni* infections, we have compiled a recombinant protein library of parasite cell-surface and secreted proteins expressed in mammalian cells.

**Results:**

Together with a time series of sera samples from volunteers experimentally infected with a defined number of male parasites, we probed this protein library to identify several markers that can detect primary infections with as low as 10 parasites and as early as 5 weeks postinfection.

**Conclusions:**

These new markers could be further explored as valuable tools to detect ongoing and previous *S mansoni* infections, including in endemic regions where transmission is low.

## Teaser

A library of recombinant *S mansoni* cell-surface and secreted proteins was produced in mammalian cells for functional and epidemiological studies. Using human and mouse sera from experimentally controlled infections, we identified molecular markers of infection as early as 5 weeks.

Schistosomiasis is a neglected tropical disease affecting more than 200 million people in 52 countries and is one of the world’s major health problems causing 200 000 deaths per year. In 2015, the impact of the disease was estimated at 3.5 million disability-adjusted life years, putting a huge socioeconomic burden on many low- and middle-income countries [1]. In humans, schistosomiasis is caused by 5 species of platyhelminth parasites belonging to the genus *Schistosoma*. Their geographical distribution is restricted by the presence of species-specific freshwater snails that act as intermediate hosts, with *Schistosoma mansoni* having the most widespread distribution, encompassing both Africa and South America [2]. Infected snails shed cercariae, a free-swimming larva, which penetrates human skin to initiate infection. Parasite maturation within its host takes several weeks: cercarial heads first remodel their surface to form schistosomula [3], which migrate through the dermis for several days before entering the host bloodstream. Eventually, young adult male and female *S mansoni* worms pair up in the liver before moving to the mesenteric blood vessels, where each pair can release over 300 eggs per day from 5 weeks postinfection [4]. The symptoms of schistosomiasis are caused by progressive accumulation of eggs within host tissues, eliciting host-derived immune responses that can eventually lead to liver fibrosis, portal hypertension, and, if left untreated, death [5]. In the absence of a licenced vaccine, treatment relies on the use of a single drug, praziquantel.

The exact prevalence of schistosomiasis worldwide may be underestimated due to limitations of routine methods of detection [6]. Diagnosis of *S mansoni* infections and subsequent decisions on mass drug administration mainly rely on observing parasite eggs in patients’ stools with the Kato-Katz test. Although this method can detect current infections, it is not sensitive enough to diagnose low levels of infection present in low-endemicity areas or in recently treated populations [7]. In these instances, detection of parasite antigens such as the circulating cathodic antigen or circulating anodic antigen (CAA) in patients’ sera or urine by sensitive field-applicable or laboratory-based assays is highly useful [8–10]. In areas where elimination has been achieved, the detection of antiparasite serum antibodies could support epidemiological surveillance that informs of any risk of resurgence. Host antibody responses to *S mansoni* parasites is often measured against whole parasite extracts such as soluble egg antigens (SEA) or soluble worm antigen preparation (SWAP), which are not molecularly defined and can lead to cross-reactivity with other helminth species. Although a diagnostic test based on a recombinant protein could mitigate these problems, only very few have been used to test patient antibody responses to *Schistosoma* infections [11]. The development of new, more sensitive diagnostic tools would therefore help to improve the detection and early treatment of schistosomiasis.

Extracellular antigens released by or displayed at the surface of the parasite at the initial stage of infection [3] can be valuable early immunodiagnostic markers because they are directly exposed to the host humoral system. The identification of such antigens has been aided by the sequencing and annotation of the *S mansoni* genome [12], and several proteomics [13–20], transcriptomics, and in silico analyses [21–23] have identified genes expressed by the schistosomula and adult worm. Despite their value, extracellular proteins pose challenges for recombinant expression because they contain structurally important posttranslational modifications such as glycosylation and especially disulphide bonds required to produce informative antibody epitopes. To address this, we have previously developed protein expression approaches in mammalian cells to compile large panels of parasite recombinant ectodomains that retain their binding activity and immunogenicity [24], enabling the identification of host-parasite receptor-ligand interactions and humoral markers of protection against malaria [25].

To identify new markers for *S mansoni* infections, we created a panel of 115 recombinant proteins representing secreted and membrane-tethered *S mansoni* proteins mostly enriched at the schistosomula stage. Using human and mouse sera from experimentally controlled *S mansoni* infections, we determined the kinetics of humoral responses to this panel of antigens in the context of primary infections and identified several early serological markers.

## MATERIAL AND METHODS

### Identification of *Schistosoma mansoni* Cell-Surface and Secreted Proteins

Genes encoding cell-surface and secreted proteins from cercarial and adult *S mansoni* were identified using published proteomic and transcriptional data [13–16, 19–21]. To further enrich for genes transcribed at the schistosomula stage, we identified 1302 transcripts upregulated at 48 versus 3 hours posttransformation [26] using EdgeR [27] as well as the 1000 most abundant transcripts at 48 hours as assessed by reads per kilobase mapped, resulting in 1977 unique genes, 274 of which encoded secreted and cell-surface proteins identified using signal peptide and transmembrane domain prediction software [28, 29]. All RNA-seq data are available in the European Nucleotide Archive (ENA) under study PRJEB3190 and run accession numbers ERR411525, ERR411535, and ERR411541 for the 3-hour time point and ERR411522, ERR411527, and ERR411538 for the 48-hour time point. Ninety-four mitochondrial, endoplasmic reticulum, or multipass proteins, which are difficult to express as a contiguous ectodomain, were subsequently excluded, leaving a short list of 180 proteins. Gene structures were manually refined by mapping transcriptome data to the genome sequence, and genes spanning gaps in the genome sequence or with ambiguity in structure were removed, resulting in a final list of 115 candidates numbered in Table 1.

**Table 1.** Details of 115 Cell-Surface and Secreted Proteins From *Schistosoma mansoni*^a^

Number	Accession No.	Name	Boundaries	MM	Domain/Protein Similarity	Level	Ref.
	Cell Surface						
1	Smp_195190	Sm13	E18-T80	37		High	[17]
2	Smp_081920	SmLy6I (Cd59.5)	L26-T104	45		Medium	[17]
3	Smp_166340	SmLy6F (Cd59.4)	L26-S98	47		Medium	[17]
4	Smp_017730	Sm200	D20-S1662	257		Medium	[14]
5	Smp_127820		V18-S760	132		Medium	[14]
6	Smp_194920		D17-T592	98	T-cell immunomodulatory protein	Low	[14]
7	Smp_011680		L30-P348	87	Cd36-like class B scavenger receptor	Low	
8	Smp_054070		D31-S210	71	TM2 domain-containing protein 3	High	
9	Smp_073400		R25-T214	78	LAMP-like protein	Medium	
10	Smp_105220	SmLyB (Cd59.2)	I20-P99	42		High	[15]
11	Smp_019350	SmLy6A (Cd59.1)	H28-T102	38		High	[15]
12	Smp_021220		E24-S119	53		High	
13	Smp_031880		R18-S240	58	Ig domain-containing protein, basigin-related	High	
14	Smp_009830		D20-S149	51	Translocon-associated protein subunit beta	Low	
15	Smp_032520		Y19-R200	74	LAMP-like protein	High	
16	Smp_074000		I19-I232	56		Low	
17	Smp_102480		N29-D66	37		High	
18	Smp_124000	MEG-14	T27-D110	38		High	
19	Smp_156270		S23-P144	47	Post-GPI attachment to protein factor	Low	
20	Smp_174580		S17-T275	59	Vesicular integral membrane protein	High	
21	Smp_176020	MEG-11	D23-P55	34		High	
22	Smp_048380		L20-S283	73		Medium	
23	Smp_060570		Y21-S428	85		Low	
24	Smp_075280		V19-T222	77	LAMP-like protein	High	
25	Smp_129840		G25-S1161	206		Medium	
26	Smp_133270		E34-T759	120	Sel1-like protein	-	
27	Smp_145420		H30-I1733	282	Plexin A3	-	
28	Smp_149740		F26-T560	130	Alzheimer disease beta-amyloid related	Medium	
29	Smp_155810		L29-T1100	184	Protocadherin 11	Medium	
30	Smp_162520		N20-P1070	162	Protocadherin fat4	Low	
31	Smp_164760		S22-P1160	184	IgSF	Low	
32	Smp_166300		N27-I356	94		High	
33	Smp_168240		Q25-T263	68		High	
34	Smp_171460		L19-S841	161	IgSF	Medium	
35	Smp_176540		V31-T956	146	Protocadherin 18	Medium	
36	Smp_157070		I19-P397	83	EGF-domain-containing protein	Low	
37	Smp_165440		Q25-P363	74	Netrin receptor unc5	Low	
38	Smp_136690		N26-T674	118	Acetylcholinesterase	Medium	[17]
39	Smp_061970		F21-P518	95	GPI ethanolamine phosphate transferase 2	-	
40	Smp_153390	SmNNP5	S19-S428	95		Medium	[14]
41	Smp_072190	SmLy6D (Sm29)	V27-T168	57		High	[14]
42	Smp_064430		A22-S155	53		Medium	[19]
	Secreted Adhesion/Growth Factor/Metabolite Binding						
43	Smp_194840		E18-I146	47	NPC-like cholesterol-binding protein	High	[50]
44	Smp_194910		N22-I180	51	Saposin B domain-containing protein	Medium	[20]
45	Smp_063530		E26-R193	49	Apoferritin	Medium	[50]
46	Smp_141680		I33-A659	110	Fasciclin domain-containing protein	High	[14]
47	Smp_043650		Q23-L81	37	Prohormone npp-28	Low	
48	Smp_170550		N27-Y928	164	Granulin	Medium	
49	Smp_035040		N24-L241	63	IgSF	Low	
50	Smp_052660		I22-R488	97	Matabotropic glutamate receptor	-	
51	Smp_128590		K21-S260	69	Laminin gamma3	Low	
52	Smp_135210		I21-Q1584	220	EGF-domain-containing protein	Low	
53	Smp_136320		H21-K154	48	GSK3beta-interacting protein	Low	
54	Smp_144130		L26-V553	118	Septate junction protein	Low	
55	Smp_154760		Q30-M2155	298	EGF-domain-containing protein	Low	
56	Smp_171780		Q19-K260	61	SPARC	High	
57	Smp_178740		Q31-V533	97	Tesmin-related protein	Low	
58	Smp_180600		S22-S292	72	IGF-binding protein	High	
59	Smp_181220		N18-I173	50	C1q-binding protein	Medium	
60	Smp_211020		L32-Y703	122	Discoidin domain-containing protein	-	
61	Smp_016490		E21-Q194	53	SaposinB domain-containing protein	Medium	
62	Smp_130100		F20-I128	45	Saposin domain-containing protein	Low	[20]
63	Smp_105420		I19-S196	53	Saposin domain-containing protein	High	
64	Smp_105450		Y19-C126	58	Saposin domain-containing protein	Medium	[50]
65	Smp_202610		V19-T135	46	Saposin domain-containing protein	Medium	
	Secreted Proteases						
66	Smp_090100		E24-Q582	98	Invadolysin	Low	[16]
67	Smp_067060		H18-N340	73	Cathepsin B1, isotype2	High	[50]
68	Smp_103610	SmCB1 (Sm31)	H18-N340	73	Cathepsin B1, isotype1	Low	[50]
69	Smp_019030		D21-L455	91	Cathepsin C/Dipeptylpeptidase 1	Low	[50]
70	Smp_002600		L17-G498	111	Lysosomal Pro X carboxypeptidase	Medium	[50]
71	Smp_071610		I24-L472	99	Dipeptidyl-peptidase II	Medium	[50]
72	Smp_089670		S19-N2127	409	Alpha-2 macroglobulin	Medium	[15]
73	Smp_112090	SmCE2a.3	F19-I263	56	Cercarial elastase 2a	-	[16]
74	Smp_119130	SmCE1a.2	W25-I263	55	Cercarial elastase 1a	-	[16]
75	Smp_002150	SmSP2	S25-F501	97	Trypsin-like serine protease	Low	
76	Smp_141610	SmCB2	N23-N347	72	Cathepsin B	Medium	
77	Smp_147730	SmKI-1	Y21-K146	47	Kunitz-type protease inhibitor	Medium	
78	Smp_034420	Sm12.8	N20-S148	56	Cystatin	Medium	
79	Smp_075800		Q20-G429	83	Hemoglobinase	Low	
80	Smp_210500	SmCL3	D17-V370	75	Cathepsin L3	Medium	
81	Smp_132480		E16-S393	79	Subfamily A1A unassigned peptidase	Low	
82	Smp_166280		C20-L337	79	Glutaminyl cyclase	Medium	
83	Smp_187 140		K21-F342	74	Cathepsin L	Low	
84	Smp_006510	SmCE2a.2	W25-I263	53	Cercarial elastase 2a	-	[19]
85	Smp_090110		E24-I591	103	Invadolysin	-	[19]
	Other Secreted Enzymes						
86	Smp_145920		Y30-F340	71	Protein tyrosine sulfotransferease	Low	
87	Smp_040790		E24-E213	54	Peptidyl prolyl cis-trans isomerase B	Medium	
88	Smp_008320		D18-D325	72	Pap-inositol-1,4-phosphatase	Low	
89	Smp_021730		G30-D220	52	Cytochrome c oxydase subunit Vb	Low	
90	Smp_078800		E25-S192	49	DnaJ subfamily B	Low	
91	Smp_007450		R22-G160	48	Heat-shock 67b2	Medium	
92	Smp_026930		T51-V442	85	Acetylglucosaminyltransferase	Medium	
93	Smp_059910		D28-Q274	70	Ser-Thr protein phosphatase	-	
94	Smp_089240		K43-Y498	93	Acetylglucosaminyltransferase	-	
95	Smp_134800		K20-T1590	242	Tyr protein kinase	Low	
96	Smp_018760		N25-L991	152	Alpha glucosidase	Low	[19]
	Secreted VALs/MEGs						
97	Smp_194860	Sm8.7	E20-E92	39		Medium	[17]
98	Smp_138080	MEG-3.1	A21-S146	50		High	[17]
99	Smp_194830	SmKK7	K21-D79	37		High	[16]
100	Smp_001890	VAL18	K27-Y194	55		Low	[16]
101	Smp_002070	VAL4	K22-E181	57		Low	[16]
102	Smp_138060	MEG-3.3	A21-G151	46		High	
103	Smp_180620	MEG-17	N17-R65	41		High	
104	Smp_123540	VAL12	I22-L24	57		Low	
105	Smp_123550	VAL8	Q24-K261	66		Medium	
	Putative Secreted Proteins						
106	Smp_181070		K25-Q114	43		Low	
107	Smp_004710		M26-S127	42		High	
108	Smp_061310		Y20-E140	65		Low	
109	Smp_005060		E17-I146	45		-	
110	Smp_141500		N27-L121	56		High	
111	Smp_006060		P35-P380	92		High	
112	Smp_063330		K24-F182	48		High	
113	Smp_096 790		T21-E94	38		Medium	
114	Smp_201730		E24-R92	38		Medium	
115	Smp_019000		D28-D236	53		Medium	[19]

Abbreviations: EGF, epidermal growth factor; GPI, glycosylphosphatidylinositol; GSK3beta, glycogen synthase kinase 3 beta; Ig, immunoglobulin; IGF, insulin-like growth factor; IgSF, Ig superfamily; LAMP, lysosome-associated membrane glycoprotein; MEGs, micro-exon genes; MM, molecular mass; NPC, Niemann-Pick C; NPP, ectonucleotide pyrophosphatase/phosphodiesterase; Ref., reference; SPARC, secreted protein acidic and rich in cysteine; VAL, venom allergen-like.

^a^For each protein, we show the accession number, alternative name, boundaries of the extracellular domain expressed in mammalian cells, expected MM in kilodaltons, domain similarities, and level of expression as determined by enzyme-linked immunosorbent assay (ELISA). References to previous proteomics studies where this protein was identified are provided where available. The expected MM of each protein was calculated by adding 3 kDa per predicted glycosylation site (the average mass of a *N*-linked glycan) to the expected mass of the protein. Levels of expression were determined from each ELISA profile as detailed under Material and Methods.

### Recombinant Protein Expression Using a Mammalian Expression System

The entire ectodomain of membrane-anchored proteins was selected, their signal peptide removed using predictions from SignalP v3.0, and the corresponding cDNAs were made by gene synthesis after codon-optimization for expression in human cells (GeneArt; Invitrogen). The ectodomains were flanked by unique *Not*I and *Asc*I restriction sites and subcloned into an expression plasmid containing the mouse V_K_7–33 signal peptide [24], and a C-terminal tag containing rat Cd4d3 + 4 domain, a BirA monobiotinylation sequence, and 6-His tag (Addgene plasmid no. 50 803) [30]. All expression constructs are available at www.addgene.org (plasmids nos. 120 590 to 120 704). Plasmids were transiently cotransfected with BirA in HEK293-E or -6E cells, supernatants were collected, and recombinant proteins were detected by Western blotting with 0.02 µg/mL streptavidin-HRP (Jackson ImmunoResearch) as previously described [24]. When proteins showed signs of proteolytic cleavages, transfections were repeated in the presence of a protease inhibitor cocktail (Sigma).

### Human Samples From Endemic Regions and Experimentally Controlled Infections

To initially characterize the immunoreactivity of the proteins, we used plasma pools from 10 nonexposed European controls and 10 Ugandan adults from a cohort living in a high transmission area [31, 32]. Individuals were selected from those who had a soluble worm antigen immunoglobulin (Ig)G1 response in the upper third for the cohort with a median egg count of 876 eggs per gram (epg) (interquartile range, 345–1967 epg). All patient samples were collected in accordance with the Uganda National Council for Science and Technology and the Cambridge Local Research Ethics Committee. Sera or plasma from volunteers experimentally infected percutaneously with 10–30 male cercariae were collected weekly in accordance with the LUMC Institutional Medical Ethical Research Committee (P16.111), as previously described [33]. All volunteers were treated with praziquantel 12 weeks after infection.

### Sera From Experimental Mouse Infections

The life cycle of the NMRI (Puerto Rican) strain of *S mansoni* was maintained by routine infections of mice and susceptible *Biomphalaria glabrata* snails under the UK Home Office Project Licence nos. P77E8A062 and PD3DA8D1F; all protocols were approved by the local Animal Welfare and Ethical Review Body (AWERB). Seven-week-old BALB/c female mice were infected percutaneously by tail immersion in water containing 200 cercariae for 40 minutes under general anaesthesia or by injection of 350 cercariae intraperitoneally. Blood samples were collected at 8, 21, and 42 days postinfection.

### Enzyme-Linked Immunosorbent Assays

Protein expression was quantified by enzyme-linked immunosorbent assay (ELISA) as previously described [24]. Briefly, serial dilutions of biotinylated proteins were captured on streptavidin-coated microtitre plates and detected by mouse antirat Cd4 OX68 antibody (AbD Serotec), followed by an alkaline-phosphatase-conjugated antimouse secondary antibody (Sigma), and proteins were classified into high (typically >5 µg/mL transfection supernatant), medium (between 1 and 5 µg/mL), and low (<1 µg/mL) levels of expression. To determine the presence of heat-labile epitopes, biotinylated proteins were captured on streptavidin-coated plates either untreated or after heat treatment for 10 minutes at 80°C before incubation with sera at 1:1000 dilution in HBST/2% bovine serum albumin (HBST/2%BSA). Statistical analysis was performed in GraphPad Prism using the Holm-Sidak method for multiple *t* tests. Sera from human volunteers or experimentally infected mice were diluted 1:250 or 1:1000, respectively, in HBST/2%BSA; binding was detected with horseradish peroxidase-conjugated antihuman or antimouse secondary antibodies (Sigma), respectively, recognizing IgA, IgM, and IgG. For each individual, the optical density (OD) value of the preinfection samples was reference-subtracted from the OD readings at all subsequent time points. The OD values for each protein were compared with that of the rat Cd4d3 + 4 tag used as a negative control, and seropositivity was defined as OD_protein_ > OD_control_ + 3SD_control_.

## RESULTS

### Selection and Expression of a Panel of 115 Secreted and Cell-Surface Proteins From *Schistosoma mansoni*

**Figure 1.**
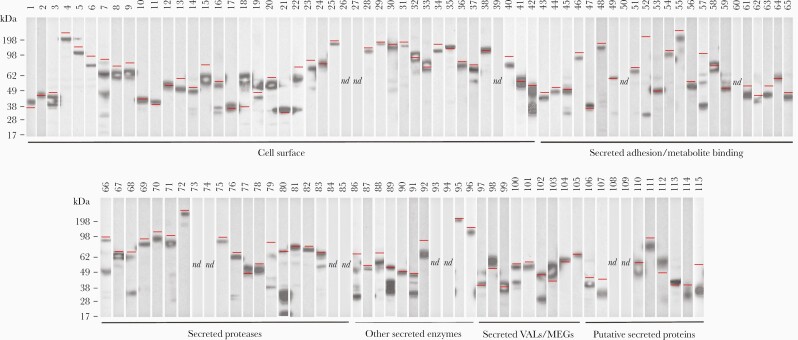
A library of 115 recombinant cell-surface and secreted proteins from Schistosoma *mansoni* expressed as secreted enzymatically monobiotinylated recombinant proteins in HEK293 cells. Supernatants were resolved by sodium dodecyl sulfate polyacrylamide gel electrophoresis (SDS-PAGE) under reducing conditions, blotted, and detected with streptavidin-conjugated horseradish peroxidase. Approximately one third of the protein library consists of membrane-tethered surface proteins, whereas the remainder of the library corresponds to secreted proteins as indicated. Their predicted molecular mass is indicated by a red line. Three proteins (52, 57, 86) migrated faster than expected; 6 proteins (42, 68, 80, 89, 91, 102) exhibited evidence of partial processing; 13 proteins were not detected by Western blotting.

To compile a library of recombinant *S mansoni* proteins, we used published proteomics data to identify 40 proteins predicted to be located on the surface or secreted by the parasite (Table 1). Because membrane and secreted proteins of the schistosomula stage were underrepresented in proteomics datasets, we supplemented our library using transcriptomics data. To identify genes transcribed early after infection, we selected 1302 transcripts enriched at 48 versus 3 hours postinfection and the 1000 most abundant transcripts in the 48-hour schistosomula. Proteins likely to be secreted or membrane-anchored were identified, resulting in a list of 274 genes. Of those, mitochondrial, endoplasmic reticulum proteins, and those with incomplete open reading frames were excluded so that 75 new candidates were added for a total of 115 genes (Table 1). One third encoded single-pass transmembrane or GPI-anchored proteins, and the remaining secreted proteins were divided into 5 broad categories (Table 1). Proteins were expressed in HEK293 cells, quantified by ELISA, and their integrity was determined by Western blotting (Table 1, Figure 1). Most proteins were detected at their expected size including the very large proteins Sm200 (257 kDa, protein 4) and α _2_-macroglobulin (409 kDa, protein 72) (Figure 1), and only 12 (10%) could not be detected (Table 1, Figure 1). This library of recombinant *S mansoni* surface and secreted proteins represents a valuable resource for immunological and functional studies.

### *Schistosoma mansoni* Recombinant Proteins Expressed in Mammalian Cells Contain Heat-Labile Conformational Epitopes

**Figure 2.**
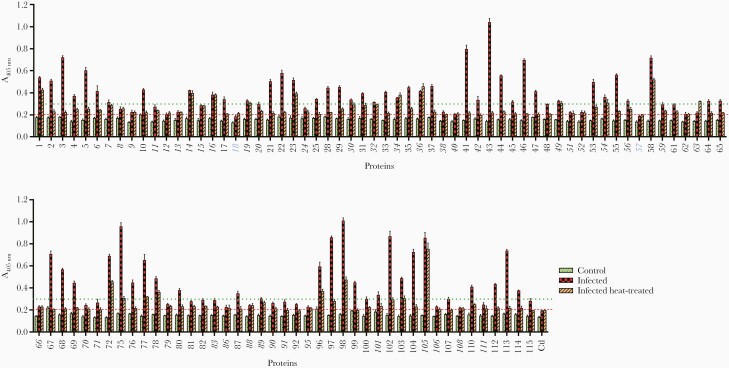
The majority of recombinant proteins are immunoreactive to sera from individuals living in schistosomiasis-endemic areas and contain heat-labile conformational epitopes. Recombinant proteins were probed with pooled sera from individuals living in schistosomiasis-endemic areas (“infected,” red checked bars) or individuals from the United Kingdom who have never been infected (“control,” green dotted bars). To test for the presence of heat-labile epitopes, recombinant proteins were also heat-treated (80°C, 10 minutes) before being exposed to immune sera (“infected heat treated,” orange hatched bars). All except 2 proteins (18 and 57, shown in blue) were seropositive, as determined by A_protein_ > A_control_ + 3SD_control_ ( = 0.201) (red dashed line), where control is the rat Cd4d3 + 4 protein tag. High immunoreactivity was determined as A_protein_ > 0.3 (green dotted line). Proteins that exhibited little or no loss of reactivity after heat treatment are shown in italics, including 16 highly reactive proteins (6, 7, 14, 16, 19, 30, 32, 34, 36, 42, 49, 54, 56, 89, 101, 105). All measurements were performed in triplicate; error bars = standard deviation.

Many antibodies elicited in the context of a natural infection recognize conformational epitopes present on the native protein. To determine the fraction of proteins containing conformational epitopes, we compared the immunoreactivity of pooled plasma from chronically exposed individuals to untreated and heat-treated proteins. All except proteins 18 and 57 were seropositive, and 66 of the expressed proteins (64%) were highly immunoreactive (A_280_ > 0.3) (Figure 2). The majority of proteins showed moderate to strong loss of immunoreactivity after heat treatment; only 16 of the highly reactive proteins showed no statistically significant loss of reactivity, suggesting that they were either natively unstructured, misfolded, or contained heat-stable domains (Figure 2). Overall, the recombinant proteins were therefore immunoreactive to plasma from individuals living in high-endemicity areas and contained heat-labile epitopes.

### Serological Profiles of Human Experimental *Schistosoma mansoni* Infections

**Figure 3.**
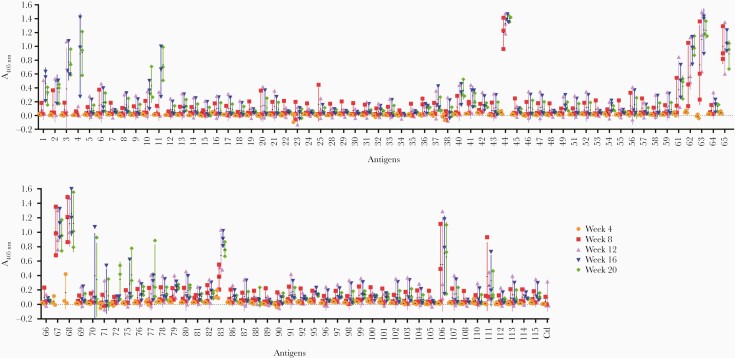
Identification of early markers of infection in human volunteers experimentally infected with *Schistosoma mansoni* male cercariae. Three individuals were each infected with 30 male cercariae, and their antibody response to 103 *Schistosoma* antigens were monitored every 4 weeks over a period of 20 weeks. The number of positive antigens increased over time with the most highly immunoreactive antigens containing saposin domains (proteins 44, 61, 62, 63, and 65) or belonging to the cathepsin family of proteases (proteins 67, 68, and 83). Colored symbols represent time point readings for each individual. Data points represent mean ± standard deviation; *n* = 3.

The recent establishment of controlled human *S mansoni* infections [33, 34] provided a unique opportunity to identify early serological markers of infection and the kinetics of the host antibody response in an experimentally controlled setting. We initially tested plasma taken at 4 weekly intervals from 3 volunteers, each infected with 30 male cercariae, against our 103 expressed *S mansoni* proteins. As expected, compared with high-endemicity plasma, fewer antigens were immunoreactive, but several were immunopositive in all participants, and the number of positive antigens increased over time (Figure 3). Five antigens (44, 63, 65, 67, and 68) showed consistently strong responses in all volunteers from 8 weeks postinfection. At 12 weeks, 4 additional antigens (61, 62, 83, and 106), which were already positive in 2 of 3 individuals at 8 weeks, were detected in all participants (Figure 3). It is remarkable that 5 of the 9 antigens seropositive by week 12 contained a saposin domain (proteins 44, 61, 62, 63, and 65), whereas proteins 67, 68, and 83 all belong to the cathepsin family of proteases. The number of antigens seropositive in all individuals increased over time up to week 20, so, in total, 20 antigens were immunoreactive in all volunteers for at least 1 time point, whereas another 17 were observed in at least 2 participants.

**Figure 4.**
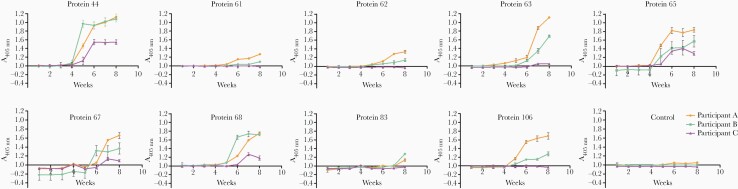
Kinetics of human antibody response to early markers of infection using sera from experimental infections by *Schistosoma mansoni*. Immunoreactivity to *S mansoni* antigens were quantified on a weekly basis in 3 individuals infected with 30 male cercariae. Reactivity to proteins 44 and 65 could be detected in all volunteers as early as 5 weeks postinfection. The control antigen corresponds to the rat Cd4d3 + 4 protein tag. Data points represent mean ± standard deviation; *n* = 3.

Early diagnosis of infection before the onset of symptoms would be very valuable; therefore, we analyzed weekly samples from all 3 individuals until week 8 against the 9 antigens positive in all volunteers at the 12-week time point (Figure 4). Immunoreactivity was observed in all volunteers as early as 5 weeks postinfection for proteins 44 and 65, 6 weeks for protein 68, and 7 weeks for proteins 63 and 67. As observed previously, only 2 of the 3 participants were reactive to proteins 61, 62, 83, and 106 between 5 and 8 weeks postinfection. In the case of antigens 44, 65, 68, and 106, seropositivity increased sharply between weeks 4 and 6 before plateauing at later time points.

**Figure 5.**
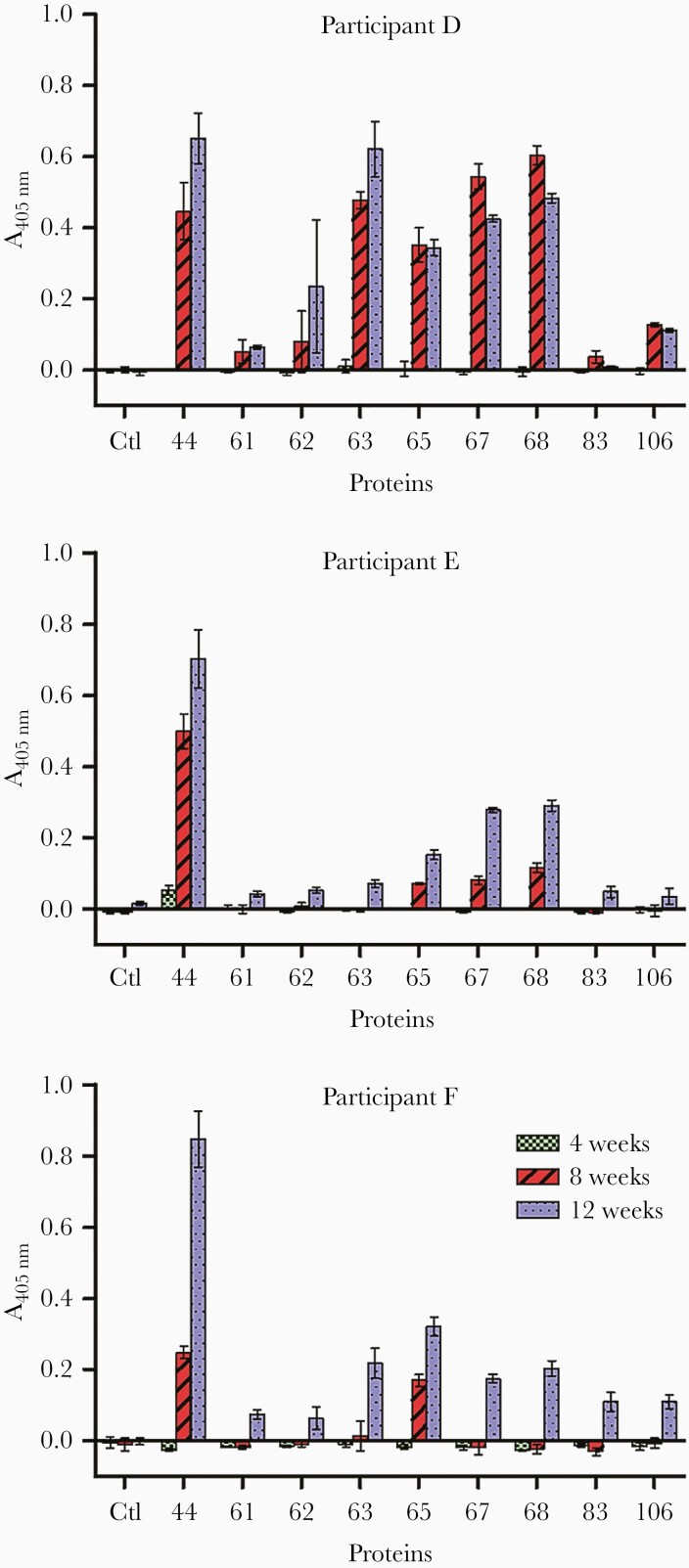
Early reactivity to *Schistosoma mansoni* antigens is detectable in individuals challenged with just 10 cercariae. The immune response from 3 human volunteers infected with 10 male *S mansoni* cercariae was monitored at 4, 8, and 12 weeks postinfection. Reactivity to antigens 44 and 65 was detected in all participants at 8 weeks and as early as 4 weeks in the case of participant E and antigen 44. With the exception of protein 83, all volunteers were immunoreactive for the antigens tested at 12 weeks postinfection. Data points represent mean ± standard deviation; *n* = 3.

To further determine the sensitivity of detection, we quantified the immunoreactivity in 3 volunteers exposed to only 10 cercariae (Figure 5). At 4 weeks, only 1 individual (participant E) was weakly seropositive for protein 44. All 3 volunteers were seropositive for proteins 44 and 65 at 8 weeks and reacted to all other antigens, except protein 83 by 12 weeks. Although immunoreactivity was lower than with the 30-cercariae dose and interindividual variability was present, proteins 44 and 65 were again the most immunogenic antigens. In conclusion, individuals experimentally infected with small numbers of *S mansoni* cercariae showed seropositivity to 5 antigens as early as 5 to 7 weeks postinfection.

### The Humoral Response Elicited by Mixed-Sex Infections in Mice Is Broadly Similar to That of Humans

**Figure 6.**
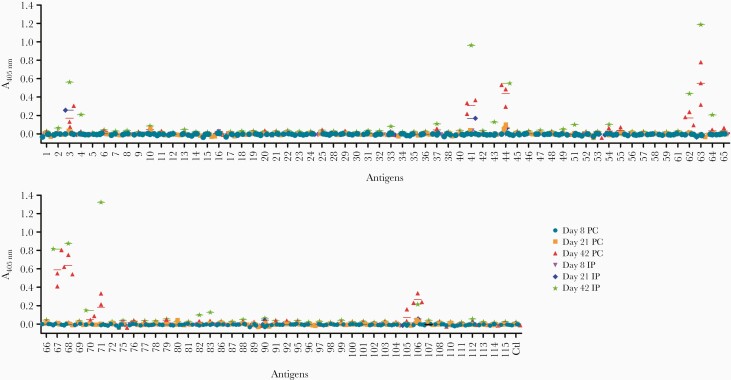
Analysis of the acquired antibody response to *Schistosoma mansoni* antigens in experimentally infected mice. Individual sera from 3 mice infected percutaneously (PC) or pooled sera from 3 mice infected intraperitoneally (IP) were analyzed at 8, 21, and 42 days postinfection. The aim was to try and capture the antibody reactivity at different stages of parasite maturation: schistosomule at 8 days, immature adult at 21 days, and mature adult at 42 days. Proteins 44 and 3 were immunopositive at day 21, and 8 additional proteins (10, 41, 62, 63, 64, 67, 68, 106) were immunopositive at day 42. Each data point represents the average of triplicate experiments; error bars = standard deviation.

Although sera from experimental infections with male carcariae identified valuable diagnostic antigens, any female-restricted antigens would not have been identified. To address this in a controlled experimental setting, we used mice as an animal model of infection. Sera were collected from mice infected either percutaneously with 200 cercariae or through intraperitoneal injection of 350 cercariae, and their serological responses were quantified (Figure 6). None of the antigens showed consistent reactivity across samples at 8 days postinfection, as expected; however, reactivity to antigen 44, and to a lesser extent, antigen 3, could be detected across all samples from day 21. At 42 days, an additional 8 antigens showed reactivity across all sera tested. Overall, reactivity with the pool of sera from mice infected intraperitoneally was stronger than individual mice infected percutaneously, possibly due to the higher number of cercariae used. Just like in human samples, proteins 44, 62, 63, 67, 68, and 106 were the most immunoreactive. In addition, reactivity to the Ly6 family members Ly6F, Ly6B, and Ly6D (proteins 3, 10, and 41, respectively) was detected at an earlier time point in mice than it was in humans. It is interesting to note that proteins 61 and 65, which were positive between 5 and 6 weeks postinfection in humans, did not react with murine samples, whereas conversely protein 71 generated a stronger response in mice.

## DISCUSSION

Despite its widespread distribution and high morbidity rate, schistosomiasis remains a neglected tropical disease whose true incidence and health impact remains underestimated [6]. In this study, we have described a recombinant protein library containing secreted and cell-surface proteins from different developmental stages of *S mansoni*, and together with sera obtained from a recently established controlled human infection model [33] we used it to determine the kinetics of the humoral response to *S mansoni* infection. Although other large arrays of parasite proteins have been used for diagnostic and immuno-epidemiological purposes, they have mostly relied on cell-free or bacterial expression systems [35, 36]. Mammalian expression systems are more suitable for the addition of structurally important posttranslational modifications found on extracellular proteins and thereby preserve conformational epitopes that can be recognized by antibodies. Using this approach, we successfully expressed 103 proteins, the majority of which were observed at their expected size. Although the 12 proteins we could not express were equally distributed among the different protein families represented in the library, 3 of them corresponded to elastases. Using pooled plasma from patients living in a high-transmission region of Uganda, we observed that 64% of the expressed proteins were strongly immunoreactive (A_280_ > 0.3) with the majority (82%) showing sensitivity to heat treatment, an indicator of tertiary folding. Although the lower immunoreactivity for 37 proteins may suggest incorrect folding, they could also be weakly immunogenic in humans or not directly exposed to the host immune system. Of the 16 most immunoreactive proteins observed, 11 had already been identified in the surface and secreted proteome of *S mansoni*; by contrast, only 6 of the 37 proteins with low serum reactivity (16%) have already been described in proteomics studies. The use of a single mammalian expression system for the production of large panels of proteins is particularly attractive for the systematic comparison of antigens in diagnostic, immuno-epidemiological, or vaccination studies, and we have used this approach previously to identify potentially protective antibodies against malaria [24, 25, 30].

Mass administration of praziquantel to schoolchildren has been the mainstay of control programs against schistosomiasis, and this has proved relatively successful in reducing parasite burdens and contributing to elimination from some areas [37]. Continued surveillance and early detection of new cases remains critical to avoid any risk of resurgence; however, the commonly used Kato-Katz method is not sensitive enough to detect low levels of parasitemia, resulting in the underestimation of the real number of cases [7]. Detection of parasite-derived glycans such as CAA in the urine or serum of patients by lateral-flow test is currently considered the most sensitive assay for the detection of current *Schistosoma* infections because it can detect very low infection levels and reactivity disappears rapidly after praziquantel treatment [8, 38]. In areas where schistosomiasis has been eliminated or is close to elimination, alternative methods of surveillance might be needed. By persisting several months or even years after infection [39], antibody responses provide a useful historical measure of parasite exposure to monitor populations at risk of resurgence. Currently, most host antibody responses are measured against crude parasite preparations such SEA or SWAP, which may suffer from considerable cross-reactivity with other helminths antigens. Therefore, species-specific recombinant proteins as diagnostic tools could be more reliable.

A striking feature of this study is the relatively small number of antigens eliciting a patent immune response in the few weeks after a primary infection. In humans, almost no IgM/IgG response could be detected before 4 to 5 weeks postinfection: some antigens might be more highly expressed at later stages of parasite development, or the parasite could evade the host immune response. Subsequently, antibody responses were almost exclusively directed at secreted proteins belonging to the saposin and cathepsin families. Of the 9 proteins reactive in all human volunteers before 12 weeks, 5 contained saposin domains and 3 were cathepsins. It is interesting to note that several saposin domain-containing proteins from *Schistosoma japonicum* have been proposed as markers of infections in mice and humans [40–42], whereas proteins 66 and 67, 2 isoforms of the cathepsin B1 protease, seem to have built-in adjuvanticity [43]. Both saposins and cathepsins are produced by the schistosome’s alimentary tract and have been involved in the digestion of lipids and proteins [44, 45]. Their reactivity to the host humoral system suggests they are produced by the parasite at an early stage. Although 5 of the 6 saposin-domain-containing proteins present in the library were immunoreactive (although reactivity of protein 83 remained modest up to 12 weeks), they share only 9% to 28% sequence identity, making cross-reactivity unlikely. The dynamics of the host response to the recombinant saposins closely parallels the detection of the parasite CAA glycans in the sera of infected patients, which is first detected at 4 weeks [38].

Reactivity to members of the uPAR/Ly6 domain-containing family, which are expressed at the somule and adult stages [46–48], was also consistently observed across human and murine samples. In our study, reactivity to Ly6F, Ly6B, and Ly6D (proteins 3, 10, and 41, respectively) was observed in both human and mouse serum samples, whereas reactivity to Ly6A and Ly6I (proteins 11 and 2, respectively) was only detected in human samples. Three of these proteins (Ly6B, Ly6D, and Ly6F) have been shown to elicit strong antibody responses in rat, mouse, and human sera [48]. Although they share some homology with the complement-inactivating Cd59 protein, they do not seem to have conserved the same function [46].

## CONCLUSIONS

By producing recombinant proteins in a mammalian expression system, we have paid particular attention to their correct folding, and thus this new resource could be used in a wide range of cellular and molecular assays such as vaccine screening [49], cellular assays looking at immunomodulatory functions, immunoepidemiological studies, or the identification of host binding partners by receptor-ligand screening. We envisage these proteins will be useful to the wider scientific community to further understand *Schistosoma* biology.

## Notes

***Acknowledgments*.** We thank Catherine McCarthy for technical help and David Dunne for helpful discussions on the study design.

***Financial support*.** This work was funded by the Wellcome Trust (Grant 206194).

***Potential conflicts of interest*.** All authors: No reported conflicts of interest. All authors have submitted the ICMJE Form for Disclosure of Potential Conflicts of Interest.

Presented in part: Joint Spring Meeting of the British Society for Parasitology, April 2019, Manchester, UK.
